# Evaluating Heart Rate Variability as a Biomarker for Autonomic Function in Parkinson’s Disease Rehabilitation: A Clustering-Based Analysis of Exercise-Induced Changes

**DOI:** 10.3390/medicina61030527

**Published:** 2025-03-17

**Authors:** Ahmed M. Basri, Ahmad F. Turki

**Affiliations:** 1 Department of Medical Laboratory Sciences, Faculty of Applied Medical Sciences, King Abdulaziz University, Jeddah 21589, Saudi Arabia; abasri@kau.edu.sa; 2 Electrical and Computer Engineering Department, Faculty of Engineering, King Abdulaziz University, Jeddah 21589, Saudi Arabia; 3 Center of Excellence in Intelligent Engineering Systems (CEIES), King Abdul Aziz University, Jeddah 21589, Saudi Arabia

**Keywords:** Parkinson’s disease rehabilitation, heart rate variability monitoring, exercise metrics analysis, remote health monitoring

## Abstract

*Background*: Heart rate variability (HRV) is a key biomarker reflecting autonomic nervous system (ANS) function and neurocardiac regulation. Reduced HRV has been associated with cardiovascular risk, neurodegenerative disorders, and autonomic dysfunction. In Parkinson’s disease (PD), HRV impairments indicate altered autonomic balance, which may be modifiable through structured exercise interventions. This study investigates the effects of aerobic exercise on HRV in patients with PD and evaluates autonomic adaptations to rehabilitation. *Methods*: A total of 110 patients with PD (55 male, 55 female) participated in a supervised three-month aerobic exercise program. HRV was assessed pre- and post-intervention using electrocardiogram (ECG) recordings. Time-domain and frequency-domain HRV metrics, including standard deviation of RR intervals (SDRR), very-low-frequency (VLF), low-frequency (LF), high-frequency (HF) power, and LF/HF ratio, were analyzed. Principal Component Analysis (PCA) and clustering techniques were applied to identify subgroups of HRV responders based on autonomic adaptation. *Results*: Significant improvements in HRV were observed post-intervention, with a reduction in LF/HF ratio (*p* < 0.05), indicating improved autonomic balance. Cluster analysis identified four distinct HRV response subgroups: Strong Responders, Moderate Responders, Mixed/Irregular Responders, and Low Responders. These findings highlight individual variability in autonomic adaptations to exercise. PCA revealed that key HRV parameters contribute differently to autonomic regulation, emphasizing the complexity of HRV changes in PD rehabilitation. *Conclusions*: This study demonstrates that aerobic exercise induces beneficial autonomic adaptations in PD patients, as reflected by HRV changes. The identification of response subgroups suggests the need for personalized rehabilitation strategies to optimize autonomic function. Further research is warranted to explore the long-term impact of HRV-guided rehabilitation interventions in PD management.

## 1. Introduction

Parkinson’s disease (PD) is the second most common neurodegenerative disorder after Alzheimer’s disease, affecting approximately 2–3% of the global population [1]. Regionally, in Saudi Arabia, a community survey by Al Rajeh et al. estimated the prevalence of PD at 27 per 100,000 [2], while a broader study by Safiri et al. reported a prevalence of 82.6 per 100,000 in the MENA region [3]. Globally, as the aging population grows, the burden of PD is projected to rise significantly, highlighting an urgent need for improved diagnostic and management strategies [4]. Both genetic and environmental factors contribute to PD, with diverse manifestations across patient populations [5]. PD is an age-related neurodegenerative condition characterized by the progressive depletion of dopamine, leading to motor and non-motor impairments [1]. Pathologically, the disease is marked by the loss of dopaminergic neurons in the substantia nigra pars compacta and the presence of Lewy bodies, cytoplasmic inclusions containing insoluble alpha-synuclein aggregates.

Clinically, PD is characterized by motor symptoms such as resting tremor, rigidity, and bradykinesia, which are considered hallmark diagnostic features [5]. However, non-motor symptoms such as anosmia, constipation, depression, and REM sleep behavior disorder often precede motor deficits by years, complicating early diagnosis [6]. In advanced stages, patients may experience cognitive decline, pain, and autonomic dysfunction [7]. Standard treatment approaches include pharmacological interventions such as levodopa-carbidopa, dopamine agonists, monoamine oxidase (MAO) inhibitors, and anticholinergics [8]. Additionally, interventional therapies such as deep brain stimulation and emerging technologies like gene therapy and ultrasonic lesioning provide options for managing advanced symptoms [9]. However, the complexity of symptom management and disease progression requires regular treatment adjustments, particularly for non-motor symptoms and motor fluctuations, which are often subjective and difficult to quantify [10].

Globally, access to specialized care for patients with PD is limited. For instance, in France, only 33.5% of patients with PD had a neurology consultation within a year, despite national guidelines recommending biannual follow-ups [11]. A similar trend was observed in Saudi Arabia, where limited public awareness, cultural stigma surrounding chronic diseases, and a lack of Arabic-language resources exacerbated delays in diagnosis and treatment [12]. These barriers not only increase the socio-economic burden on patients and healthcare systems but also highlight the need for innovative and accessible solutions to bridge the gap in care.

One promising approach to overcoming these challenges is the use of heart rate variability (HRV) as a biomarker for autonomic nervous system function [13]. Since PD is associated with progressive autonomic dysfunction, assessing HRV changes may provide a quantifiable measure of disease severity and response to interventions [13]. Furthermore, HRV can be monitored non-invasively, making it a practical tool for remote or resource-limited clinical settings, where frequent neurological assessments may not be feasible [14].

Heart rate regulation is a finely tuned process governed by the autonomic nervous system (ANS), where the sympathetic and parasympathetic branches dynamically modulate cardiac activity [13]. Beat-to-beat fluctuations in heart rate, known as heart rate variability (HRV), serve as a direct physiological marker of autonomic function and adaptability [14]. Changes in sympathetic and vagal tone influence these fluctuations, making HRV a valuable tool for assessing autonomic balance [14].

Standardized HRV metrics, derived from R–R intervals on electrocardiograms (ECG), allow for the quantitative assessment of autonomic activity, following established consensus guidelines [15]. Time-domain measures, such as the standard deviation of R to R (SDRR) intervals, provide direct insights into overall HRV, while frequency-domain measures, obtained via spectral analysis (typically using Fast Fourier Transform), offer a more detailed breakdown of autonomic influences [15].

Within the frequency domain, different spectral components correlate with distinct autonomic mechanisms [15]:

- Total power represents overall autonomic modulation.
- Low-frequency (LF) activity (<0.15 Hz) is primarily linked to baroreflex-mediated autonomic regulation, incorporating both sympathetic and parasympathetic influences.
- High-frequency (HF) activity, on the other hand, is strongly associated with parasympathetic (vagal) activity, reflecting respiratory-related heart rate fluctuations.
- The LF/HF ratio is often interpreted as an index of autonomic balance, though this measure remains debated due to factors such as differences in temporal dynamics between sympathetic and parasympathetic responses and variations in cardiac pacemaker sensitivity.

HRV is increasingly recognized as a non-invasive biomarker for neurological function and brain health, particularly in neurodegenerative diseases such as Parkinson’s disease (PD) [16]. Autonomic dysfunction is a well-documented non-motor symptom of PD, with HRV abnormalities emerging early in the disease process, often preceding motor impairments [17].

HRV changes in patients with PD reflect underlying neurodegeneration within autonomic regulatory pathways, including the following [18]:

- Degeneration of brainstem autonomic centers (e.g., dorsal motor nucleus of the vagus, locus coeruleus, and medullary cardiovascular centers), leading to impaired parasympathetic regulation.
- Dysfunction of basal ganglia and limbic circuits, which play a role in autonomic modulation and stress regulation.
- Reduced baroreflex sensitivity and vagal tone, contributing to blood pressure variability, orthostatic hypotension, and cardiovascular instability.

Studies have demonstrated that HRV reductions in patients with PD correlate with disease severity, suggesting its potential as a quantitative biomarker for tracking disease progression and autonomic deterioration [19]. Additionally, exercise-based interventions have been shown to improve HRV parameters, reflecting enhanced autonomic resilience and neural plasticity in patients with PD undergoing structured rehabilitation [20].

Recent advances in artificial intelligence (AI) and machine learning (ML) have expanded the potential of HRV analysis for personalized rehabilitation strategies in PD. AI-driven models offer the ability to predict individual responses to exercise-based interventions. Santilli et al. (2024) demonstrated how ML algorithms can assess rehabilitation outcomes in neurological patients, suggesting that how AI-driven rehabilitation can enhance patient independence, reduce complications, and optimize care [21]. AI-enhanced monitoring could refine patient classification and optimize exercise prescriptions based on predicted autonomic responses [21].

Furthermore, neural network models have been applied to predict motor and proprioceptive responses in rehabilitation by analyzing complex sensory–motor interactions [22]. These AI-driven techniques could be adapted to HRV-based rehabilitation, allowing for real-time autonomic function tracking and predicting individualized treatment efficacy [21]. By integrating unsupervised clustering with AI-assisted HRV modeling, rehabilitation protocols can be further optimized for improved autonomic and neurocardiac adaptation in patients with PD [22].

This study aims to assess the feasibility of using heart rate variability (HRV) as a biomarker for autonomic function changes in patients with Parkinson’s disease (PD) undergoing structured exercise interventions. By analyzing pre- and post-intervention HRV metrics, this study evaluates the impact of exercise on autonomic regulation and explores HRV’s potential as a quantitative measure for rehabilitation effectiveness in PD.

## 2. Materials and Methods

### 2.1. Participants

This study involved 110 participants (55 males and 55 females) diagnosed with Parkinson’s disease (PD), aged 60.2 ± 4.5 years, with a BMI of 28.5 ± 2.8 kg/m^2^. Participants were selected based on strict inclusion and exclusion criteria established by the coordinating medical team to ensure safety and suitability for the study.

#### 2.1.1. Inclusion Criteria

Participants were included if they met the following criteria:

- Aged between 45 and 75 years;
- Had a diagnosis of Parkinson’s disease, classified as Hoehn and Yahr stages 2–3, indicating moderate disease severity;
- Were stable on PD medications for at least three months prior to the study;
- Could walk independently or with minimal assistance (Unified Parkinson’s Disease Rating Scale—Motor Examination, score ≤ 3 on gait and posture items);
- Had not participated in any structured exercise program or physical activity regimen (e.g., more than three sessions per week, 30–40 min per session) in the past six months or longer;
- Were able to understand and follow instructions in either Arabic or English.

#### 2.1.2. Exclusion Criteria

Participants were excluded if they met the following criteria:

- Had contraindications to magnetic resonance imaging (MRI), such as metallic implants, claustrophobia, or pacemakers;
- Had significant medical, neurological, or psychiatric conditions unrelated to Parkinson’s disease (e.g., recent strokes, uncontrolled diabetes, or major depressive disorder);
- Displayed severe cognitive impairments (Montreal Cognitive Assessment (MoCA) score < 21), which could hinder their ability to follow instructions;
- Experienced advanced motor complications (Hoehn and Yahr stage > 3) that could compromise their safe participation;
- Were classified as obese (BMI ≥ 30.0 kg/m^2^), as excessive weight may impact autonomic function, HRV measurements, and exercise tolerance.

Furthermore, the exclusion of patients with MRI contraindications, such as metallic implants, pacemakers, or claustrophobia, was essential to ensure accurate HRV measurements, methodological consistency, and participant safety. Pacemakers and deep brain stimulators can introduce electromagnetic interference, distorting ECG signals and confounding HRV analysis. Additionally, Parkinson’s patients with implanted devices often have altered autonomic responses due to cardiovascular comorbidities, making it difficult to isolate exercise-induced HRV changes. Although MRI was not directly used in this study, excluding these individuals ensures compatibility with potential future neuroimaging research. Furthermore, patients with claustrophobia or anxiety disorders could experience autonomic dysregulation unrelated to exercise, further skewing HRV findings. Therefore, this exclusion criterion enhances data reliability, maintains a standardized study population, and upholds ethical considerations.

These inclusion and exclusion criteria were designed to ensure that participants had moderate PD severity, could safely engage in rehabilitation activities, and provided meaningful data for analysis.

### 2.2. Data Collection

In the study, heart rate variability (HRV) data collection was conducted, and the Biopac MP 150 ECG system (BIOPAC Systems, Goleta, CA, USA) was employed to record electrocardiograms. Data acquisition and processing were facilitated by the National Instrument NI-USB-6128 (National Instruments, Austin, TX, USA), which performed analog-to-digital conversion. This setup was complemented by a custom-made LabView program running on an HP ProBook 640 G8 (Hewlett-Packard, Palo Alto, CA, USA) laptop for managing the data collection.

The measurement protocol involved baseline recordings taken over a period of 5 min while participants were in a supine position to ensure consistency across measurements. After these initial measurements, participants engaged in a prescribed exercise program in the lab to show them how to perform the prescribed physical exercises.

Over the next three months, users performed prescribed exercises that were provided by the patient’s care team through smart fitness trackers.

At the end of the three-month period, participants returned to the lab, where post-intervention measurements were again taken using the ECG system to compare changes over the duration of the exercise program.

The exercise program for Parkinson’s Disease (PD) patients was carefully designed to enhance motor function, cardiovascular health, and overall well-being while accounting for disease-specific challenges such as tremors, rigidity, bradykinesia, and balance impairment. Utilizing a custom developed Adaptive Digital Exercise Coach (ADEC) system (Center of Excellence in Intelligent Engineering Systems (CEIES), Jeddah, Saudi Arabia), which integrated a wearable fitness tracker and a digital monitoring platform, the program ensured patient engagement, adherence, and progress tracking. The prescribed regimen consisted of aerobic exercise training (AET) to improve cardiovascular endurance, strength and resistance training (SRT) to enhance muscle strength and prevent falls, balance and coordination exercises (BCEs) to improve postural stability, and flexibility and stretching (FS) routines to reduce stiffness and maintain joint mobility. The intensity, duration, and frequency of exercises were gradually increased based on individual physical capabilities and compliance data. Sessions began with 20 min of exercise three times a week, progressing by the fourth week to 150 min per week, distributed across either 30 min sessions five times a week or 50 min sessions three times a week, always including a 5 min warm-up and cool-down. The exercise selection was adapted to the patient’s mobility level, incorporating activities such as walking, stationary cycling, resistance bands, and chair-based exercises, ensuring accessibility across different stages of PD. To ensure a structured approach, all prescribed exercises shared key characteristics. Intensity levels were gradually adjusted, beginning at 50–60% of the patient’s maximum heart rate and increasing to 70–80% over time, with adjustments made based upon baseline HRV measurements taken from the initial ECG clinic session. The duration of sessions was systematically increased to allow for progressive adaptation while preventing overexertion. The type of exercise was tailored to minimize fall risk while maximizing functional benefits, prioritizing low-impact exercises such as walking and cycling, alongside targeted strength training, balance drills, and dynamic stretching to address specific PD symptoms. Adherence was closely monitored through various compliance metrics, including session completion rates, attendance tracking, duration adherence, and intensity adherence. The ADEC system’s smart tracking features provided real-time monitoring of the patient’s progress. The fitness tracker’s heart rate sensors continuously recorded heart rate, while motion data from accelerometers, gyroscopes, and barometric altimeters tracked step count, movement patterns, and elevation changes. The system automatically recorded exercise start and end times, transmitting these data to a secure cloud-based server for clinician review. Compliance metrics were analyzed in real time, including duration compliance (percentage of prescribed exercise completed, averaging 85.32%), heart rate compliance (percentage of time spent in the target heart rate range, averaging 60.28%), and momentary heart rate compliance (ensuring the target HR was reached at least once per session). To enhance adherence, patients received automated reminders and motivational messages, while a clinician portal allowed healthcare providers to monitor adherence and adjust exercise prescriptions as needed. Real-time visual and haptic feedback during workouts further supported patients in maintaining their target exercise intensity. Overall, the exercise program for patients with PD was designed to be progressive, personalized, and adaptable, ensuring safe and effective training while preventing overexertion. By leveraging the ADEC system’s real-time tracking and clinician oversight, the program significantly improved adherence and patient outcomes, empowering individuals with PD to maintain physical activity, mobility, and independence.

### 2.3. Statistcal Analysis

The data processing phase included the detection of QRS complexes using a MATLAB, R2022 b program (MathWorks, Natick, MA, USA) specifically designed to identify R wave peaks from the ECG data. The time between these successive peaks, known as Interbeat Intervals (IBIs) or RR intervals, was accurately measured.

(1) $${RR}_{i}=t_{R_{i}}-t_{R_{i-1}}$$

where

${RR}_{i}$: The *i*-th RR interval (time between successive heartbeats, in milliseconds);

$t_{R_{i}}$: Time of occurrence of the R-peak at beat *i*;

$t_{R_{i-1}}$: Time of occurrence of the previous R-peak at beat *i* – 1.

In addition, to ensure the accurate and smooth interpolation of heart rate variability (HRV) signals, we employed cubic spline interpolation, a method that fits multiple cubic polynomials between successive data points while preserving continuity in the first and second derivatives. This technique provides a refined approach to handling missing or unevenly sampled data by generating a smooth curve that best represents the underlying physiological trends.

The general cubic spline function for the interval [*x_i_*, *x_i+_*_1_] is defined as follows:

(2) $$S_{i}\left(x\right)=a_{i}+b_{i}\left(x-x_{i}\right)+c_{i}(x-x_{i}{)}^{2}+d_{i}(x-x_{i}{)}^{3}\;\;\;\;,\;x_{i}\leq x\leq x_{i+1}$$

where

$S_{i}\left(x\right)$: the interpolated RR interval at time *x*;

$x_{i}$ and $x_{i+1}$: the time points at which RR intervals were originally sampled;

$a_{i}$: the RR interval (*RR_i_*) at $x_{i}$, which represents the baseline measurement at that specific point;

$b_{i}$: the first derivative (slope) at $x_{i}$, ensuring continuity in HRV trends;

$c_{i}$ and $d_{i}$: the second and third derivative coefficients, respectively, which maintain smoothness and curvature consistency across intervals.

HRV analysis was conducted using both time-domain and frequency-domain measures. Time-domain analysis included calculating the mean, standard deviation, and coefficient of variation in the IBI. HRV metrics were extracted from both baseline (pre-intervention) and exit (post-intervention) visits to serve as clustering features. The selected parameters included time-domain measures such as HR standard deviation (HR SD) and HRV coefficient of variation (HRV CV), as well as frequency-domain measures including VLF, LF, HF, and the LF/HF ratio.

(3) $$SDRR=\sqrt{\frac{1}{N}\sum_{i=1}^{N}({{RR}_{i}-\bar{RR})}^{2}}$$

where

*SDRR*: Standard deviation of RR intervals (ms);

*N*: Total number of RR intervals in the given time period;

*RR_i_*: *i*-th RR interval (ms);

$\bar{RR}$: Mean RR interval over the analyzed period (ms).

(4) $${SDRR}_{Coeff.}=\frac{SDRR}{\bar{RR}}\times 100$$

where

${SDRR}_{Coeff.}$: Coefficient of variation in RR intervals (%).

For the frequency-domain measures, Power Spectral Density (PSD) analysis was conducted through Fourier Transform to assess the power within VLF, LF, and HF bands.

(5) $$PSD\left(f\right)={\left|ℱ(RR\left(t\right))\right|}^{\;2}$$

(6) $$P_{band}=\int_{f_{1}}^{f_{2}}PSD\left(f\right)df$$

where

*PSD(f)*: Power Spectral Density at frequency;

$ℱ(RR\left(t\right))$: Fourier Transform of the RR interval time series;

$P_{band}$: Total power in a specific frequency band;

$f_{1},$ $f_{2}$: Lower and upper frequency limits of the band.

To assess autonomic balance, the ratio of low-frequency (LF) to high-frequency (HF) power was computed as follows:

(7) $$\frac{P_{LF}}{P_{HF}}$$

where

$P_{LF}$: Power in the low-frequency (LF) range (0.04–0.15 Hz);

$P_{HF}$: Power in the high-frequency (HF) range (0.15–0.4 Hz);

$P_{LF}/P_{HF}$: Ratio of low-to-high frequency power, an indicator of autonomic balance.

To classify patients into distinct HRV-based response groups, an unsupervised clustering approach using *k*-means clustering was employed using Python software version 3 (Python Software Foundation, Wilmington, DE, USA). This method allowed for the identification of natural subgroups within the dataset based on pre- and post-intervention HRV characteristics. Patients were categorized into HRV-based subgroups using the *k*-means clustering algorithm, which minimizes the within-cluster variance:

(8) $$J=\sum_{k=1}^{K}\sum_{i\epsilon C_{k}}||x_{i}-\mu_{k}|{|}^{2}$$

where

*J*: Cost function (Within-Cluster Sum of Squares, WCSS);

*K*: Number of clusters;

$x_{i}$: HRV feature vector of patient *i*;

$C_{k}$: Set of patients assigned to cluster *k*;

$\mu_{k}$: Centroid (mean feature vector) of cluster *k*;

$||x_{i}-\mu_{k}|{|}^{2}$: Squared Euclidean distance between patient *i* and the centroid of its cluster.

The analysis focused on understanding variations in autonomic function among patients with Parkinson’s disease (PD) undergoing rehabilitation.

To ensure robust clustering results and statistical reliability, missing HRV values were systematically addressed before analysis. Missing data primarily resulted from ECG signal artifacts, involuntary movements in the patients with Parkinson’s disease (PD), and transient physiological irregularities that affected the accurate detection of R-peaks. Given that clustering analysis relies on complete datasets to effectively differentiate patient subgroups, we applied listwise deletion to exclude cases with missing values, preventing biased or distorted cluster formation. To further enhance comparability, Z-score standardization was applied to all HRV parameters, ensuring that differences in scale across HRV metrics did not disproportionately influence clustering outcomes. This transformation standardized each feature to have a mean of zero and a standard deviation of one, allowing for equitable contribution of all HRV parameters in PCA and clustering models. Importantly, less than 5% of the dataset contained missing values, and no entire patient records were excluded, preserving the statistical integrity of the study. This approach ensured that our clustering-based subgroup identification accurately reflected exercise-induced autonomic changes in patients with PD without introducing analytical biases.

To determine the optimal number of clusters, the Elbow Method was applied by analyzing the Within-Cluster Sum of Squares (WCSS) across a range of cluster values (*k* = 2 to 10). Additionally, the Silhouette Score was used to evaluate the quality of cluster separation, with results indicating that *k* = 4 provided the best balance between distinct subgrouping and meaningful separation. Following this, *k*-means clustering (*k* = 4) was applied to the standardized dataset, assigning each patient to one of the four clusters.

For visualization, PCA was conducted to reduce the high-dimensional HRV dataset to two principal components (PC1 and PC2). The first component, PC1, captured the majority of the variance in HRV, likely reflecting overall autonomic function improvements post-intervention. The second component, PC2, accounted for additional variability, particularly changes in spectral HRV measures. A PCA scatter plot was generated to visually distinguish patient subgroups based on clustering results.

The study adhered to high ethical standards, with approval from the Institutional Review Board (IRB) of the Center of Excellence in Intelligent Engineering Systems at King Abdulaziz University (approval number 24-CEIES-Biomed-2024). All participants provided written informed consent before participation, ensuring compliance with ethical guidelines. Furthermore, this study was conducted in accordance with the ethical principles outlined in the Declaration of Helsinki, ensuring that participants’ rights, safety, and well-being were prioritized throughout the research process.

## 3. Results

### 3.1. Heart Rate Variblity Changes Pre- and Post-Exercise

**Figure 1:**
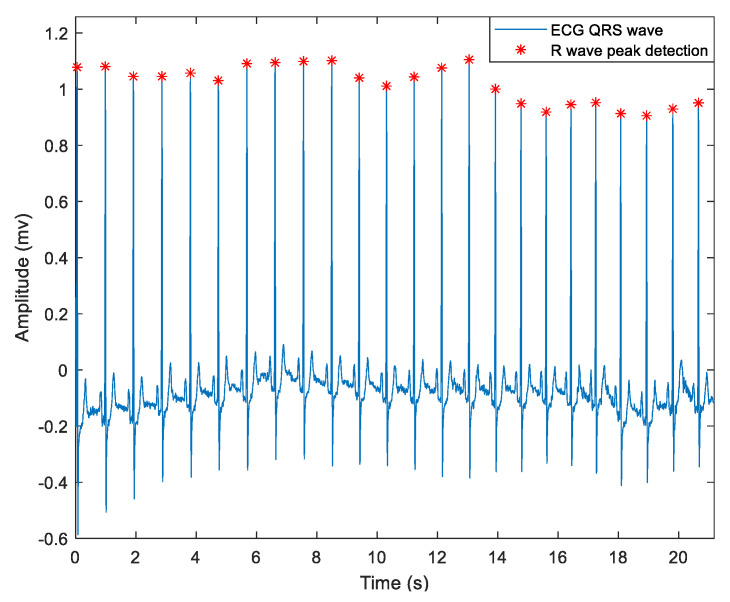
ECG signal of one subject, with QRS wave detection shown in blue and identified R-peaks marked with red asterisks.

Artifact removal and signal interpolation techniques were applied to ensure accurate peak detection and minimize noise interference. Figure 1 illustrates a representative ECG segment with detected R-peaks, highlighting the method used for RR interval extraction.

**Figure 2:**
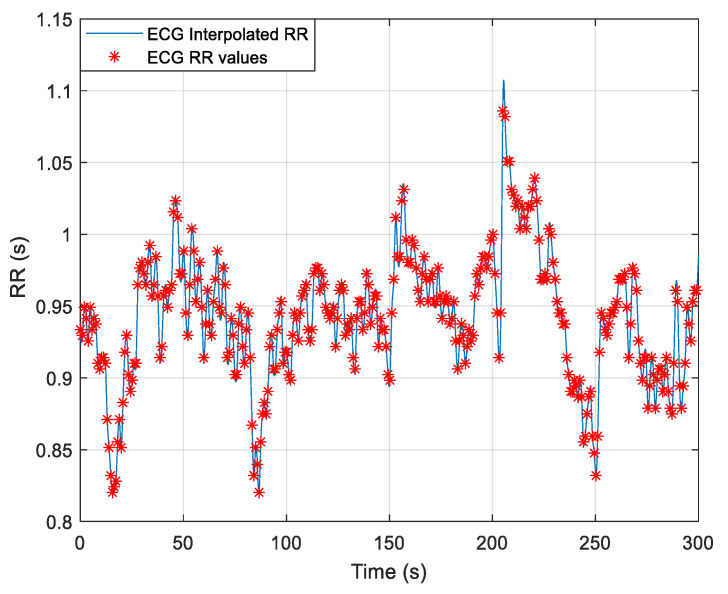
The RR interval values extracted from the ECG signal of a subject, where raw RR values are marked with red asterisks, and the interpolated RR curve is shown in blue.

Figure 2 illustrates the raw RR intervals (red markers) detected from the ECG signal, alongside the interpolated RR curve (blue line), which was used for further HRV feature extraction.

The following sections present the results of HRV pre- and post-exercise changes, highlighting key findings from time-domain and frequency-domain analyses. Additionally, unsupervised clustering was performed to classify patients into distinct HRV-based response subgroups, allowing for a deeper understanding of individual variability in autonomic adaptation to exercise therapy.

### 3.2. Frequency-Domain Analysis

**Figure 3:**
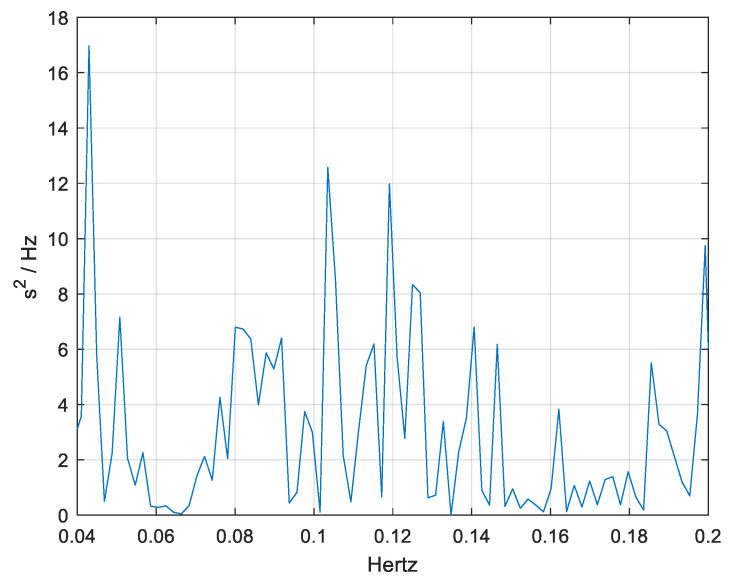
The pre-intervention Power Spectral Density (PSD) analysis of heart rate variability (HRV) for one of the participants.

**Figure 4:**
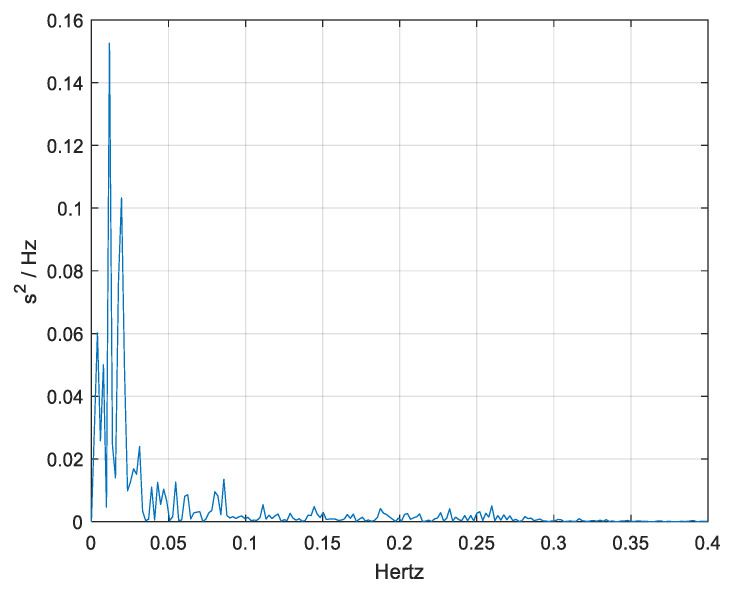
Post-intervention Power Spectral Density (PSD) analysis of heart rate variability (HRV) for one of the participants following the structured exercise program.

Figure 3 and Figure 4 represent the pre-intervention and post-intervention PSD for one of the participants, providing insights into the distribution of HRV power across different frequency bands.

Figure 4 illustrates the PSD distribution after exercise, providing insight into the modulation of autonomic nervous system activity in response to rehabilitation. Compared to the pre-intervention PSD, there is a noticeable reduction in overall spectral power, particularly in the LF and VLF bands, suggesting a shift in autonomic balance. The reduction in LF power may indicate a decrease in sympathetic dominance, while the attenuation of VLF components suggests potential stabilization of longer-term regulatory mechanisms. These findings align with previous studies indicating that aerobic exercise enhances parasympathetic activity and reduces sympathetic overactivity in Parkinson’s disease patients. By comparing pre- and post-intervention PSD trends, we can assess the degree of autonomic adaptation to structured exercise therapy, providing valuable insights into the potential role of HRV as a biomarker for rehabilitation effectiveness in PD.

### 3.3. HRV-Based Evidence of Autonomic Adaptation

The results presented in Table 1 below highlight significant reductions in the SD of RR intervals (*t*-test: *p* < 0.05) and LF/HF ratio (*t*-test: *p* < 0.05) post-intervention, suggesting a shift toward improved autonomic balance with reduced sympathetic dominance. Meanwhile, VLF power also decreased significantly (*t*-test: *p* < 0.05), indicating the potential stabilization of long-term regulatory mechanisms. These findings suggest that exercise therapy contributes to enhanced parasympathetic modulation and autonomic flexibility in Parkinson’s disease patients. The detailed HRV comparisons are summarized in the following table.

**Figure 5:**
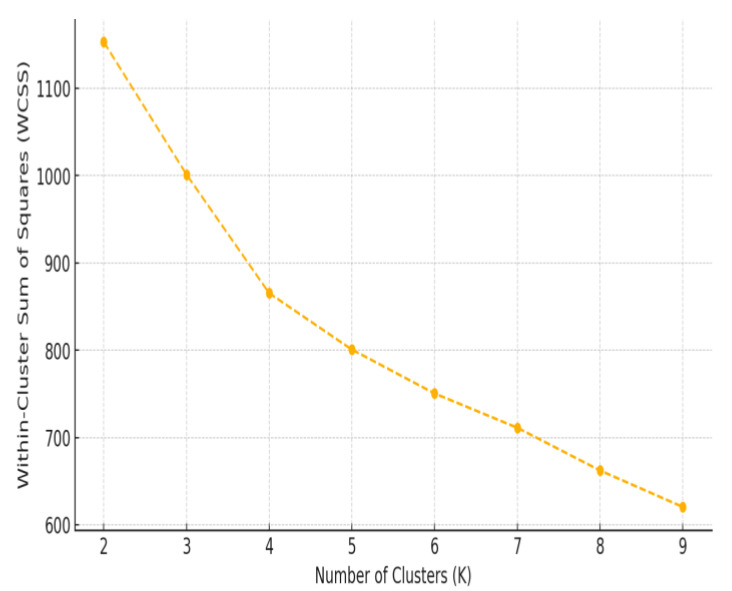
Elbow Method for optimal cluster selection in HRV-based patient segmentation.

Figure 5 displays the Elbow Method plot, where a sharp decline in WCSS is observed initially, followed by a more gradual decrease as *k* increases. The point where the curve begins to flatten—resembling an “elbow”—suggests the optimal number of clusters. In this case, the elbow appears around *k* = 4, indicating that dividing the dataset into four clusters provides a balance between minimizing variance and avoiding overfitting.

This data-driven clustering approach ensures that HRV-based patient subgroups are formed based on physiological similarities, enabling more targeted analysis of autonomic function responses to exercise intervention.

**Figure 6:**
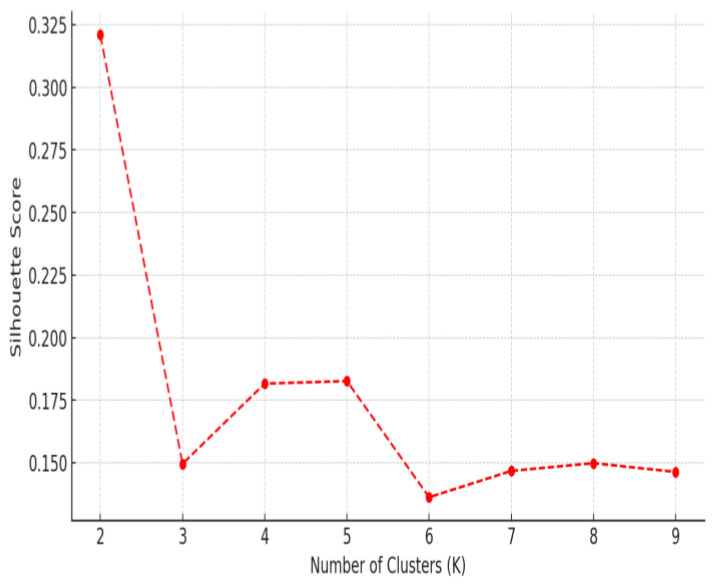
Silhouette Score analysis for optimal clustering of HRV-based patient subgroups.

Figure 6 presents the Silhouette Score for different values of *k* (number of clusters). The highest score is observed at *k* = 2, suggesting that two clusters provide the most distinct separation. However, after *k* = 3 and *k* = 4, the Silhouette Score remains relatively stable before declining at *k* > 5, indicating that adding more clusters beyond this point does not improve the clustering quality.

Given that the Elbow Method suggested *k* = 4 and the Silhouette Score stabilizes at *k* = 4, we selected *k* = 4 clusters for the final analysis. This decision ensures a balance between distinct subgrouping and capturing meaningful HRV variations across patients.

Based on the *k*-means clustering analysis (*k* = 4), Parkinson’s disease patients were grouped into four distinct HRV-based subgroups, reflecting different patterns of autonomic adaptation to exercise intervention. These subgroups were classified according to their pre- and post-intervention HRV responses, allowing for a deeper understanding of individual variability in autonomic function changes.

The largest group, Cluster 3 (Mixed/Irregular Responders), comprised 60% of the patients and exhibited high variability in HRV response, with some individuals showing partial improvements while others displayed no clear trends. Cluster 1 (Moderate HRV Responders), representing 27% of the patients, demonstrated measurable HRV changes, though their overall autonomic improvements remained moderate.

A small subset of patients, Cluster 2 (Strong HRV Responders), accounted for only 6% of the cohort but exhibited the most pronounced improvements in HRV metrics, suggesting a strong autonomic response to exercise rehabilitation. In contrast, Cluster 0 (Low HRV Responders), also representing 6% of patients, showed minimal or no improvements in HRV, potentially indicating underlying autonomic dysfunction or more advanced PD severity. Table 2 summarizes the distribution of patients across these HRV-based subgroups.

**Figure 7:**
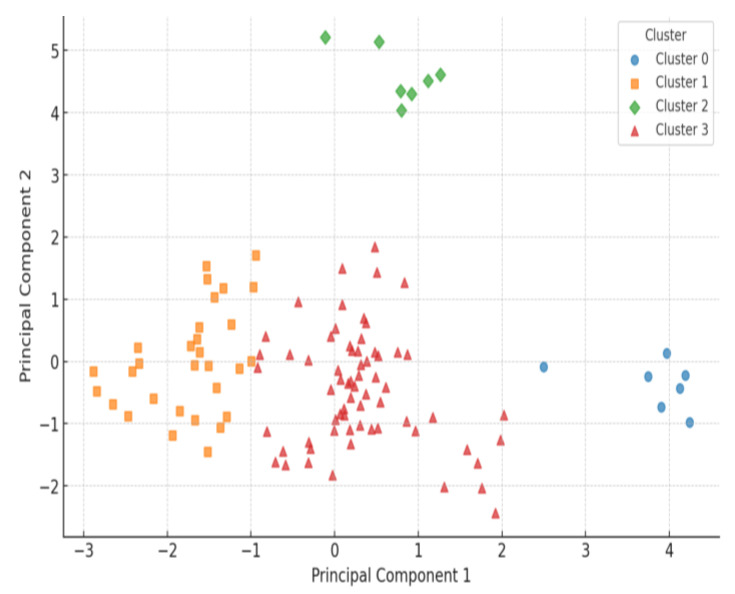
Clustering of patients with Parkinson’s disease based on their heart rate variability (HRV) response to exercise intervention.

The scatter plot in Figure 7 represents the distribution of HRV-based patient subgroups, obtained using PCA for dimensionality reduction. Each point corresponds to an individual patient, with different symbols denoting the four identified clusters. The separation between clusters highlights distinct HRV response patterns, reinforcing the validity of the subgroup classification.

The horizontal axis (PC1) captures the primary source of variance in HRV changes, while the vertical axis (PC2) accounts for secondary variations in autonomic function. The distribution of clusters confirms the distinct separation of HRV response patterns, with Cluster 2 (Strong HRV Responders) positioned at the higher end of PC2, indicating strong autonomic improvements, while Cluster 0 (Low HRV Responders) is isolated on the right, suggesting minimal HRV changes. Cluster 1 (Moderate Responders) and Cluster 3 (Mixed/Irregular Responders) show more overlapping distributions, reflecting a range of intermediate HRV responses.

## 4. Discussion

This study investigated the effects of structured aerobic exercise on HRV in patients with PD, revealing significant autonomic function changes and distinct patient subgroups with varying HRV responses. The findings suggest that exercise-based rehabilitation may serve as a non-pharmacological strategy to enhance autonomic regulation in PD but also highlight the heterogeneous nature of autonomic dysfunction and individual differences in treatment response.

### 4.1. Impact of Exercise on Autonomic Function

The observed increase in the SD of the RR intervals (SDRR) post-intervention suggests an enhancement in autonomic flexibility, a crucial marker of cardiovascular and neurological health. Autonomic dysfunction in PD is characterized by reduced HRV and impaired vagal activity, which has been linked to disease progression, increased cardiovascular risk, and poor quality of life [23]. The significant reduction in LF/HF ratio post-exercise indicates a shift away from sympathetic overactivity toward improved parasympathetic modulation, reinforcing previous evidence that exercise enhances vagal tone in neurodegenerative conditions [24].

A key finding was the significant decrease in VLF power post-intervention, which may indicate the stabilization of long-term autonomic control mechanisms. VLF power is influenced by thermoregulation, hormonal activity, and inflammatory responses [15], which are often altered in PD. A reduction in VLF power may reflect improved homeostasis and reduced autonomic instability, suggesting that exercise contributes to systemic physiological regulation beyond cardiovascular adaptation alone [25].

Interestingly, LF and HF power did not show statistically significant post-exercise differences, which could indicate individual variability in short-term autonomic adaptation. Previous studies have reported mixed findings regarding LF and HF power responses to exercise, possibly due to differences in exercise intensity, session duration, or baseline autonomic dysfunction in patients with PD [26,27,28]. Given that the LF/HF ratio significantly decreased, the balance between sympathetic and parasympathetic activity appears to have improved, even though absolute LF and HF values varied across individuals.

### 4.2. Heterogeneous HRV Responses: Patient Subgroup Differences

Unsupervised clustering revealed four distinct HRV-based patient subgroups, underscoring the variability in autonomic adaptation to exercise therapy. The largest group, Cluster 3 (Mixed/Irregular Responders, 60%), exhibited highly variable HRV responses, suggesting that while some patients experienced partial autonomic improvements, others showed inconsistent changes, possibly due to fluctuating disease states, medication effects, or underlying autonomic dysfunction.

Cluster 1 (Moderate HRV Responders, 27%) demonstrated clear but moderate improvements in HRV, indicating that some patients experience predictable autonomic benefits from exercise but may require longer or higher-intensity interventions to maximize gains.

The smallest group, Cluster 2 (Strong HRV Responders, 6%), exhibited the most pronounced improvements in autonomic function, suggesting that certain patients with PD retain significant autonomic plasticity, enabling them to respond robustly to exercise-based rehabilitation. Understanding why these patients benefit more—whether due to genetic predisposition, early disease stage, or superior baseline autonomic function—could help optimize rehabilitation strategies for a broader population [29].

Conversely, Cluster 0 (Low HRV Responders, 6%) showed minimal or no improvements, raising important questions about barriers to autonomic adaptation. This subgroup may include patients with advanced PD, severe dysautonomia, or comorbidities that limit autonomic recovery. Future interventions targeting alternative exercise modalities (e.g., resistance training, neuromuscular stimulation, or breathing exercises) could be explored to assess whether different rehabilitation strategies may yield more effective outcomes in this group.

This visualization further supports the clustering validity, demonstrating how unsupervised learning effectively distinguished patient subgroups based on HRV dynamics.

### 4.3. Clinical and Physiological Implications

The findings reinforce the importance of personalized rehabilitation strategies for PD patients. While some individuals demonstrate clear autonomic improvements, others experience minimal or inconsistent changes, highlighting the need for individualized exercise prescriptions based on baseline HRV characteristics.

Moreover, HRV could serve as a biomarker for assessing rehabilitation effectiveness in PD. Identifying pre-intervention HRV profiles that predict strong responses to exercise therapy may enable clinicians to tailor interventions more effectively, optimizing treatment outcomes and resource allocation.

The observed changes in VLF, LF/HF ratio, and SDNN also align with the broader cardiovascular health improvements seen in exercise studies involving neurodegenerative populations [30]. Since autonomic dysfunction is a predictor of cardiovascular risk in PD, improving HRV through exercise may have long-term benefits beyond motor symptom management, potentially reducing hypertension, arrhythmias, and sudden cardiac events, which are elevated in patients with PD [31].

Additionally, the absence of significant LF and HF power changes in some patients highlights a potential need for multimodal therapy approaches. Combining exercise with pharmacological modulation (e.g., acetylcholinesterase inhibitors, beta-blockers), behavioral interventions (e.g., meditation, cognitive training), or non-invasive brain stimulation (e.g., vagus nerve stimulation, transcranial direct current stimulation) could enhance autonomic responses and further improve cardiovascular regulation in patients with PD [32].

### 4.4. Limitations and Future Directions

While this study provides valuable insights into HRV as a biomarker for autonomic function in PD rehabilitation, several limitations must be considered. First, the relatively small sample size may limit the generalizability of the findings, emphasizing the need for larger-scale studies to confirm these results. Second, potential selection bias exists as the study focuses on individuals at Hoehn and Yahr stages 2–3, excluding those with more advanced motor and autonomic dysfunctions, which may influence rehabilitation outcomes.

Future studies should incorporate broader patient cohorts to improve applicability. Additionally, the short duration of the intervention (three months) restricts conclusions about the long-term sustainability of autonomic improvements. Extended follow-ups are necessary to assess whether HRV changes persist over time. The absence of a control group also limits the ability to isolate the effects of exercise from other influencing factors, such as medication adjustments or lifestyle changes.

A randomized controlled trial (RCT) would provide stronger causal evidence. Moreover, external variables such as medication use, sleep quality, stress, and diet were not fully controlled in this study, all of which could affect HRV.

Advances in wearable sensor technology and AI-driven HRV analysis have further expanded the potential of HRV as a real-time monitoring tool, allowing for continuous autonomic assessment in clinical and home settings [21,22,33]. For PD patients, integrating HRV tracking with personalized rehabilitation programs could provide valuable insights into treatment efficacy, disease progression, and overall nervous system health [21,22,34].

With the growing availability of low-cost sensors and wearable technologies, HRV assessment is now becoming increasingly feasible, enabling real-time autonomic function monitoring across diverse patient populations [35].

This expanding technological landscape presents an opportunity for the wider clinical adoption of HRV-based diagnostics, particularly in conditions where autonomic dysfunction plays a central role [34]. Further research into HRV’s predictive capabilities in Parkinson’s disease, cognitive impairment, and neurodegenerative disorders will be essential in fully harnessing its potential as a biomarker for brain health and autonomic function monitoring.

With advancements in sensor accuracy and data analytics, wearable devices offer a practical and scalable solution for long-term PD management in remote and home-based settings [36]. The International Movement Disorder Society (MDS) has acknowledged their potential, forming a task force to explore the diagnostic and therapeutic applications of wearable technology in movement disorders [36]. The UK National Institute for Health and Care Excellence (NICE) has also published recommendations for integrating wearable devices into PD care, particularly for treatment monitoring [36].

Future research should incorporate detailed patient logs to minimize confounding effects. Lastly, the study relied on ECG-based HRV analysis, which, while precise, is not always feasible for long-term real-world monitoring. While wearable devices offer continuous HRV tracking, their accuracy requires further validation before clinical implementation [36]. Despite these limitations, this study provides a foundation for HRV-based monitoring in PD rehabilitation, underscoring the need for future research to validate HRV as a clinical biomarker and refine its role in personalized PD management.

## 5. Conclusions

This study explored the feasibility of using heart rate variability (HRV) as a biomarker for autonomic function changes in patients with Parkinson’s disease (PD) undergoing structured exercise interventions. The findings demonstrate that exercise-induced HRV modifications can provide valuable insights into autonomic adaptations and rehabilitation effectiveness. Time-domain and frequency-domain HRV metrics revealed significant changes post-intervention, suggesting an improvement in autonomic regulation and parasympathetic activity following structured aerobic exercise.

Additionally, unsupervised clustering analysis identified distinct patient subgroups based on HRV response patterns, highlighting individual variability in autonomic adaptation. This reinforces the potential of HRV-based monitoring for personalized rehabilitation strategies tailored to each patient’s autonomic function.

Despite the study’s limitations, the results support HRV as a promising tool for tracking autonomic function in PD rehabilitation. Future research should focus on long-term HRV monitoring, validation of wearable-based HRV tracking, and integrating machine learning approaches to enhance predictive accuracy.

## Figures and Tables

**Table 1:** Participant demographics and HRV Metrics pre- and post-intervention with principal component analysis loadings.

Demographics					
Age (years)			60.2 ± 4.5		
Sex			55 Female + 55 Male		
BMI (kg/m^2^)			28.5 ± 2.8		
HRV Metric	Time	ECG (Mean ± SD)	*t*-test *p*-value	PCA Loadings (Feature Importance)	
				PC1	PC2
SD of RR (ms)	Pre-Intervention	3.4 ± 1.17	<0.05	−0.45	0.35
	Post-Intervention	4.02 ± 1.56		0.20	0.59
Mean of RR (ms)	Pre-Intervention	882 ± 13	0.5	-	-
	Post-Intervention	890 ± 15		-	-
SDRR Coeff. of Variation %	Pre-Intervention	46 ± 17	<0.05	−0.36	0.32
	Post-Intervention	41 ± 21		0.18	0.58
VLF (ms2)	Pre-Intervention	0.56 ± 0.62	<0.05	−0.02	−0.06
	Post-Intervention	0.38 ± 1.44		0.08	0.13
LF (ms2)	Pre-Intervention	0.45 ± 0.68	0.40	−0.15	−0.05
	Post-Intervention	0.37 ± 0.61		0.14	0.22
HF (ms2)	Pre-Intervention	0.16 ± 0.09	0.33	−0.12	−0.09
	Post-Intervention	0.20 ± 0.26		−0.04	0.09
LF/HF	Pre-Intervention	2.81 ± 2.30	<0.05	0.58	−0.01
	Post-Intervention	1.85 ± 8.78		0.44	0.00

**Table 2:** HRV-based patient subgroups identified through clustering.

Cluster	Subgroup Name	Number of Patients (%)
Cluster 3	Mixed/Irregular Responders	66 (60%)
Cluster 1	Moderate HRV Responders	30 (27%)
Cluster 2	Strong HRV Responders	7 (6%)
Cluster 0	Low HRV Responders	7 (6%)

## Data Availability

The data presented in this study are available on request from the corresponding author. The data are not publicly available for privacy reasons.
